# Accumulation of Trace Metals in Fruits from Mango and *Syzygium guineense* Growing in Residential Households from a Contaminated District of Lubumbashi (DR Congo): Is Fruit Consumption at Risk?

**DOI:** 10.3390/toxics11070620

**Published:** 2023-07-17

**Authors:** Serge Langunu, Precis Mpia Imanda Imabo, Benie Bibi Fwanda, Jacques Kilela Mwanasomwe, Gilles Colinet, Mylor Ngoy Shutcha

**Affiliations:** 1 Ecology, Ecological Restoration and Landscape, Faculty of Agronomic Sciences, Université de Lubumbashi, Lubumbashi 1825, Democratic Republic of the Congo; precympia12@gmail.com (P.M.I.I.); blessingfwanda4@gmail.com (B.B.F.); jacqueskilela@gmail.com (J.K.M.); mylor.ngoyshutcha@unilu.ac.cd (M.N.S.); 2 Water-Soil-Plant Unit, TERRA Gembloux Agro-Bio Tech, University of Liege, 5030 Gembloux, Belgium; 3 Plant Ecology and Biogeochemistry, Université Libre de Bruxelles, 1050 Bruxelles, Belgium

**Keywords:** Lubumbashi, trace metal, pollution, accumulation, safe weekly consumption

## Abstract

Copper smelting has been a source of soil contamination with trace metals in Penga Penga (Lubumbashi). The residents are exposed to trace metal ingestion, and planting trees is challenging in such soil conditions. Nevertheless, planting trees in former household dumps or using various types of amendments has allowed the provisioning of fruits in a few residences. From the perspective of scaling up the process, a survey has been conducted with the aim of assessing the effectiveness of the planting processes on the trace metal content in fruits and leaves of *Mangifera indica* L. and *Syzygium guineense* (Willd) DC. Samples were collected from residential households in Penga Penga and Kalebuka (a non-polluted suburb). The bioconcentration factor (BCF) and the safe weekly consumption (SWC) were calculated for each species. The results showed higher values of total and soluble concentrations of Cu, Pb, and Zn in the rhizosphere of the two species in Penga Penga. Metal concentrations were higher in the fruits and leaves from Penga Penga, with 47% of samples above the FAO and WHO thresholds (vs. 18.5% in Kalebuka). The BCF values were below 1, demonstrating the effectiveness of the process in reducing the translocation of metals to leaves and fruits. Recommendations from the SWC limit Pb consumption to 9 kg for mango flesh and Cd consumption to 6.6 kg for *S. guineense* fruits in Penga Penga (vs. 78 kg and 68 kg in Kalebuka). Finally, the results of this study provide interesting lessons for the scaling up and technical itinerary of planting trees in Penga Penga.

## 1. Introduction

More than a century of mining activity has severely impacted the environment in and around major towns of the Katangese Copperbelt (KCB) in the southeastern DR Congo [1,2]. Indeed, many publications have reported evidence of the impacts of mining activities on the trace metal pollution of the atmosphere [3], rivers [4,5,6,7], and soil [8,9,10]. Numerous sites are affected by trace metal pollution in Lubumbashi. However, the pollution in the Penga Penga district by emissions from the copper smelter of the Gécamines Lubumbashi plant is one of the oldest and best documented. In this area, the deposition of trace-metal-rich particles after the emission of fumes by the smelter has resulted in high concentrations of trace metals in soils, as well as their acidification [3,9,11,12,13], with a footprint spreading over more or less 30 km in the direction of the prevailing winds [14]. The pollution generated has led to the replacement of the original open forest with a short, sparsely vegetated landscape with large patches of bare soil [11,15]. These high levels of pollution have exposed populations in the KCB to trace metals [16] with significant health impacts [17,18]. In recent years, the situation has been exasperated by uncontrolled urbanization and population growth in Lubumbashi [19,20], including polluted areas like Penga Penga. 

In this context, phytoremediation trials have been implemented during the past 15 years in order to suggest strategies that could reduce population exposure. The results have highlighted the strong abilities of local copper flora in the phytoremediation of polluted soils in Penga Penga [13,21]. Nevertheless, anarchic urbanization and population growth have exacerbated the complexity of implementing a large-scale phytoremediation program. Indeed, the presence of residential households is a barrier to revegetating large continuous areas and leads to additional expectations regarding the establishment of vegetation in the district, as reported by Mwanasomwe [22]. In particular, the residents have clearly indicated the need to plant woody species in order to improve local living conditions through the reduction in dust by windbreaks, the creation of microclimates (e.g., creation of shade), and the production of fruits and leaves that are edible or used in traditional medicine. Unfortunately, growing trees in polluted soils is challenging for most of the residents, who are declared to have a high mortality rate. Indeed, only 6.5% of the plots (44 out of 674) have at least one tree [22].

Observations in the site have shown that residents have managed to grow trees in Penga Penga by planting them in locations of former household dumps or by using various types of organic and inorganic amendments such as soils from termite mounds (personal observations) [22]. Fruits and leaves can be directly consumed or used for healthcare in traditional medicine, respectively. Previous studies have not highlighted the risks of ingesting the fruit and leaves of trees in this neighborhood, nor have they associated the conditions in the rhizospheres with the concentrations found in the parts consumed. In addition, the World Health Organization (WHO) has established thresholds for the safe consumption of foods based on their trace metal concentrations [23]. In practice, it represents the maximum amount of trace metals that can be safely ingested by the population without any kind of intoxication or related health issues. In the context of high and multiple metal pollution in Penga Penga [12,21,24], recommendations need to be made on the amount of fruit and leaves that can be safely consumed by residents. The trace metals that limit consumption will also have to be identified in order to contribute to the implementation of technical itineraries that favor the low accumulation of metals in the aerial parts of the trees, thereby reducing human exposure. 

In this context, the general objective of this paper is to evaluate whether the tree planting protocols followed in Penga Penga have influenced the trace metal content of fruits and leaves and hence consumer safety. The specific objectives were: (i) to assess the conditions of concentrations and mobility of trace metals in the rhizosphere of the trees grown in Penga Penga, in comparison to an unpolluted district (Kalebuka); (ii) to determine the accumulation of metals in the fruits and leaves of *Mangifiera indica* and *Syzygium guineense;* and (iii) to recommend the amounts to be safely consumed by residents. The results obtained from this study are needed for the establishment of a technical protocol for producing fruits and leaves while limiting the accumulation of metals and reducing the risks of contamination of the food chain. 

## 2. Materials and Methods

### 2.1. Study Sites

**Figure 1:**
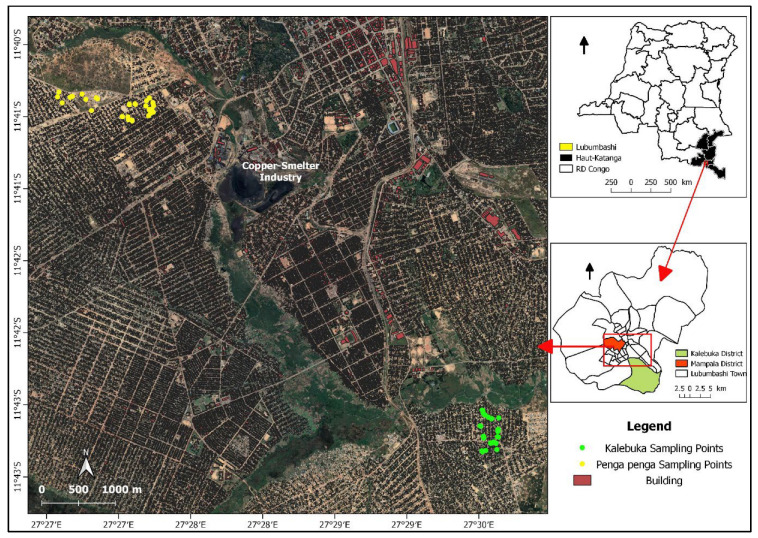
Location of the study area and sampling sites in Penga Penga and Kalebuka.

The study was carried out in two districts of Lubumbashi (Figure 1): Penga Penga and Kalebuka. They are both located in the south of Lubumbashi and are relatively similar in age (around 20 years). They result from the “anarchic” extension of Lubumbashi and its huge demographic growth observed since the first decade of the 21st century. Unfortunately, Penga Penga is located in an area polluted by the former emissions from the Gécamines Copper Smelter, with total metal concentrations (mg.k^−1^) of the order of: Cd = 6.5–8.5; Co = 109–384; Cu = 3524–50,000; Pb = 249–2657; and Zn = 290–5900, accompanied by an acid pH (4.6–5.5) [24]. Kalebuka is located in the opposite direction, where the influence of fumes from the smelter has been much lower. Table 1 presents the trace metal concentrations (after extraction with ammonium acetate + EDTA) in the surface soil of the two districts.

Lubumbashi is characterized by a climate that alternates a rainy season (November to April) and a dry season (May to October). The average annual rainfall is around 1200 mm, while the average annual temperature is 20 °C with lower temperatures in the first part of the dry season (5–16.5 °C at night) and maximum temperatures recorded in October (33 °C to 39 °C).

A previous study [22] has shown a greater density and diversity of trees in Kalebuka compared to Penga Penga. However, the most abundant fruit tree species in both areas are mango (*Mangifera indica* L.), avocado (*Persea americana* L.), and guava (*Psidium guajava* L.). *Syzygium guineense* is the most frequent native woody species in both districts, although the number of individuals remains very low compared to the exotic species [22].

Observations in both settlements have shown that planting had mainly been performed in holes amended with household wastes. However, Mwanasomwe [22] reported that people in Penga Penga use a greater variety of amendments and larger holes than those in Kalebuka. The size of the planting holes in plots where the trees have good vigor is more than one square meter wide and fifty centimeters to one hundred centimeters deep in Penga Penga. The trees considered were at least ten years old.

### 2.2. Soil and Plant Sampling

Soil samples were collected from the rhizospheres of *M. indica* and *S. guineense* in Penga Penga and Kalebuka in November and December 2021, on days without rain, between 9 a.m. and 1 p.m. In each district, soil samples were taken from the rhizosphere of 15 individuals of each of the two species, for a total of 30 samples per district and 60 soil samples for the whole study. At each sample point, four samples were collected from a 0–20 cm depth within a distance of 1 m from the tree trunk. These four samples were carefully mixed to produce a composite sample. In all, three soil samples were taken per day. The collected samples were placed in polythene bags, labeled, and dried at room temperature (<35 °C) for 10 days. They were then crushed and sieved through a 2 mm sieve and sent to the laboratory for chemical analysis. 

Leaf and fruit samples were taken from the same trees as the soil samples, for three fruit and leaves samples per species per day. Leaves were selected from those that showed no apparent signs of disease or pest attack. At least ten leaves were collected from each tree to make a composite sample. For the fruits, six to ten fruits were collected per tree to form a composite sample. As with the leaves, the fruits were selected from those that showed no apparent signs of disease or pest attack. A total of 30 leaves and 30 fruit composite samples of *M. indica* and *S. guineense* were collected from each district. They were washed with a 1% alconox solution according to the protocol proposed by Faucon et al. [25]. They were then oven-dried at 105 °C for 24 h, mixed, and ground. 

### 2.3. Chemical Analysis of Plants and Soil

Analyses were performed at the laboratory of the Soil Water Exchange Axis of Gembloux Agro Bio-Tech (Université de Liège, Liege, Belgium). The soil pH was measured potentiometrically in the supernatant with a glass electrode pH meter, after stirring 5 g of soil in 50 mL of CaCl_2_ (0.01 M) solution for 2 h, followed by centrifugation at 3000 rpm for 10 min. The total concentrations of Al, Fe, Ca, K, Mn, Cu, Pb, and Zn were determined by a portable XRF using a Titan S2 (Bruker, Glasgow, UK). The measurements of total Cd and Co concentrations were not satisfactory by XRF. Soluble concentrations were determined after extraction with 0.01 M CaCl_2_ in the solution used for the measurement of pH after filtration through filter paper (595 ½). As, Cd, Co, Cu, Pb, and Zn were measured using an Agilent 5100 ICP-OES (Agilent Technologies, Santa Clara, CA, USA). The limits of detection (mg.kg^−1^) were 0.01 for As and Cd, 0.04 for Co and Cu, 0.06 for Zn, and 0.16 for Pb.

For plant samples, mineralization was carried out using 65% HNO_3_ and 75% HClO_4_. Concentrations of As, Cd, Co, Cu, Pb, and Zn were determined by atomic absorption spectrometry (AAS, VARIAN 220, Agilent Technologies, SANTA Clara, CA, USA) [26]. It should be noted that the pulp of the fruits of *M. indica* and *S. guineense* were not separated from the skin, as consumption by the population is with the pulp. 

### 2.4. Calculation of Trace Metal Bioconcentration Factors

The bioconcentration factor (BCF) considers the bioaccumulation of metals in plants and the concentration of trace elements in the soil. It was used herein for predicting the concentration of Cu, Pb, and Zn in the leaves and fruits of the targeted species [27,28]. It is calculated as follows [29]:

Bioconcentration factor = C_tissue_ of plants/C_soil_

where C _plant tissue_ is the concentration of the metal leaves or fruits (mg.kg^−1^) and C_soil_ is the concentration of the metal (the total concentration) in the soil (mg.kg^−1^).

### 2.5. Determination of the Safe Weekly Consumption (SWC)

The safe weekly consumption (SWC) was calculated on the basis of data available in the literature and the concentration values of the trace metals in the fruit. The calculations were conducted for a 60 kg person. The following formula was used for the calculation:

SWC = SWI × C

where SWC = safe weekly concentration (no health risk) of the food (fruit); SWI = safe weekly intake of the metal (= amount of metal that can be ingested without health risk); and C = concentration of the metal in the food (fruit).

The SWI value was obtained from the values available in the literature, including recommendations from the FAO and WHO [23] and from Pelkonen et al. [30]. These values are presented as tolerable consumption per day (PMTDI), per week (PTWI), or per month (PTMI). They have been recalculated on a weekly basis for consistency (Table 2).

Furthermore, the recommendations for the amount of fruit to be consumed per week are based on the most limiting elements, i.e., those for which the lowest SWC values have been observed.

The SWC was not calculated for mango and *S. guineense* leaves as they are not subject to direct consumption as a vegetable or salad but rather used for the preparation of teas for consumption in traditional medicine.

### 2.6. Statistical Analysis

A normality test was applied to the data on soil and plant parameters. As the distributions were not normal, even after Box–Cox transformations, the non-parametric test of Mann–Whitney (MW) was used to compare concentrations between Penga Penga and Kalebuka and between mango and *S. guineense*. To compare the rhizosphere of the two species, the Mann–Whitney test was performed separately in each district. The proportion of leaves and fruits with values above the limit recommended by the FAO and WHO [23] was calculated by dividing the number above the limit by the total number of samples. All statistical analyses were performed using R Studio 3.1.

## 3. Results

### 3.1. Soil Mineral Composition in the Tree Rhizosphere 

The Ca concentration and pH were higher (*p* < 0.05) in the tree rhizosphere in Penga Penga compared to Kalebuka (Table 3). With the exception of Al_2_O_3_ (%), where there is a significant difference, there was no significant difference for Fe_2_O_3_ (%), and K. As expected, the total concentrations of As, Cu, Pb, and Zn were higher in Penga Penga. The average total Cu concentration was seven times higher in Penga Penga (1379 mg.kg^−1^) compared to Kalebuka (189 mg.kg^−1^).

The maximum values of total concentrations recorded in Kalebuka were higher than the range of values reported for natural forest soils in the Lubumbashi region. Regarding soluble contents, the Cu concentration was higher in Penga Penga (1.4 mg.kg^−1^) compared to Kalebuka (0.7 mg.kg^−1^). There were no significant differences for the other elements.

There was a significant difference between the rhizospheres and the surrounding soils in Penga Penga (not shown). The Ca concentrations and pH were higher in the rhizospheres, while the As, Co, Cu, Pb, and Zn total concentrations were higher in the surrounding unamended soil.

### 3.2. Accumulation and Bioconcentration Factors of Trace Elements in Plants in Penga Penga and Kalebuka

As, Pb, and Zn were in higher concentrations (*p* < 0.05) in the leaves obtained from Penga Penga, while there was no difference for Cd (both species) or Cu (*S. guineense*). All trace metals were higher in concentration (*p* < 0.05) in fruits harvested in Penga Penga.

The MW test applied to each site separately showed varying trends. In Penga Penga, *M. indica* had higher concentrations (*p* < 0.01) of As, Cu, Co, Pb, and Zn in leaves and higher concentrations (*p* < 0.01) of Cd, Cu, and Pb in fruits. The fruits of *S. guineense* showed higher concentrations of Cd and Zn. In Kalebuka, *M. indica* had higher Cd concentrations in leaves and fruits. It also had higher concentrations of As and Zn in leaves. *S. guineense* had higher concentrations of Cu in leaves and fruits (Table 4).

**Figure 2:**
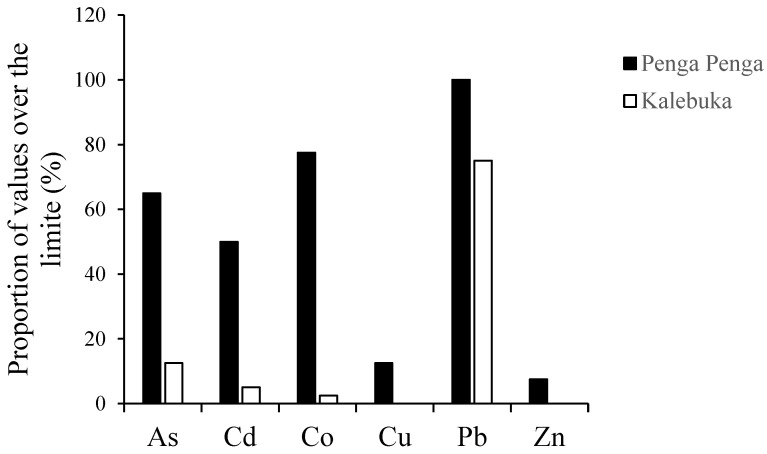
Proportion of concentration values above the WHO limit for fruits and vegetables. Limit according to the FAO and WHO [23].

The results also show that 47% of the concentration values recorded in Penga Penga were above the FAO and WHO limits vs. only 18.5% in Kalebuka. The highest proportion of values above the limit was recorded with Pb (in both districts) (Figure 2). All of the Pb concentration values recorded in Penga Penga (leaves and fruits) were above the FAO and WHO limits (0.03 mg.kg^−1^). In Kalebuka, all of the Pb values in leaves and 50% of the values in fruits were above the limit. Zn and Cu were the two trace metals with the lowest proportions of values above the recommended limit. Only 7.5% and 12.5% of the Zn and Cu concentrations were above the limit in Penga Penga, while all of the values of these two trace metals were below the limit in Kalebuka.

**Figure 3:**
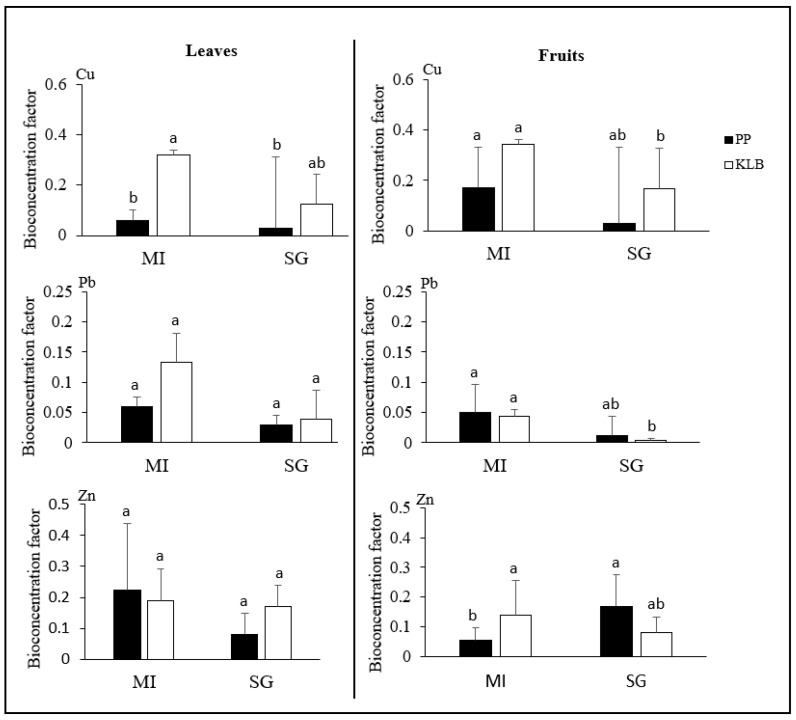
Bioconcentration factor of Cu, Pb, and Zn in leaves and fruits of *M. indica* (MI) and *S. guineense* (SG) (*n* = 60). On the left, BCFs in leaves and on the right BCFs in fruits Penga Penga (PP) and Kalebuka (KLB). Values are represented by the mean ± standard deviation; means with the same letter are not significantly different (*p* < 0.05)*; n* represents the number of samples.

All bioconcentration factor values (leaves and fruits) were below one in both districts (Figure 3). In general, it can be seen that the BCFs were higher in Kalebuka compared to Penga Penga. For Cu, the BCF values for leaves and fruits were always higher in Kalebuka. However, there were exceptions for *M. indica* in leaves and *S. guineense* in fruits, whose BCFs were slightly higher for Zn in Penga Penga. The BCF values were similar for all species and in both sites for Pb.

### 3.3. Safe Weekly Consumption (SWC)

Table 5 presents the results of the calculation of the safe weekly consumption of fresh fruits for a 60 kg person according to the SWC of each metal. Consistent with the concentration values in the fruit, the SWC values are generally higher in Kalebuka compared to Penga Penga. However, the results show that the limiting elements for fruit consumption of the two species in Penga Penga are Pb (for mango) and Cd (for *S. guineense*). These two trace metals limit the average consumption of mango flesh (including the skin) to 9 kg per week and *S. guineense* fruit to 6.6 kg per week.

In Kalebuka, the results recommend average consumptions of 68 kg and 78 kg per week of *S. guineense* and mango fruits, respectively, according to Co and Cu concentrations. 

## 4. Discussion

### 4.1. Trace Metal Concentration in the Tree Rhizosphere

As expected, the results showed higher trace metal concentrations in the rhizosphere in Penga Penga compared to Kalebuka (Table 3). The deposition of trace-metal-rich particles contained in the smoke emitted by the smelter chimney of the Gécamines plants in Lubumbashi explains the high concentrations observed in Penga Penga [21,24], as the study area is located in the pollution cone under the influence of the prevailing winds [14]. However, it can be seen that the samples collected from the rhizosphere present contrasting conditions compared to those reported for the surrounding soils (Table 1). This is most likely explained by the use of amendments with neutralizing properties such as termite mound soil [31]. The high calcium concentration observed from the rhizosphere supports this hypothesis due to the well-known action of Ca oxides and hydroxides on increasing pH values [32,33]. Shutcha et al. [21] have already reported higher pH values in soil samples collected under *Microchloa altera* populations established on former landfill sites, as well as under *Setaria pumila* populations established on former stripped termite mounds in Penga Penga. Secondly, the total trace metal concentrations were found to be lower than in the surrounding unamended soil in Penga Penga. Indeed, total concentrations above 50,000 mg.kg^−1^ have been recorded in Penga Penga [12,34], whereas the maximum in the rhizosphere was below 4800 kg^−1^ (Table 3). Nevertheless, it can be seen that the concentrations of metals in the rhizosphere in Penga Penga remain very high (on average six times higher) compared with references in the region [9]. This can be explained by two reasons: the high concentrations of trace metals in the amendments used and/or a lateral diffusion of metals from the surrounding soils to the rhizospheres. Indeed, residents of Penga Penga use a wide range of soil amendments when planting trees to ensure their survival and performance (personal observations), including termite mound soils [22]. However, high concentrations of metals have been reported in the surface soils of termite mounds located in Penga Penga [35]. Regarding metal transfer from the surrounding soils, Mwanasomwe et al. [36] reported similar trends from tree plantations installed on tailings at the Kipushi hydrometallurgical plant (30 km south of Lubumbashi). Other studies have reported these phenomena, which are attributed to water movements and the subsequent diffusion of mineral elements in soils [37]. This result demonstrates the value of the long-term monitoring of plantations established on soils enriched in trace metals, since it indicates the enrichment of the rhizosphere over the years.

Finally, it was very interesting to note the low proportion of soluble trace metal fractions (extractable with 0.01 M CaCl_2_) in the rhizospheres of both species in Penga Penga, with Pb and Zn values similar to those observed in Kalebuka (Table 3). This low availability of trace metals is most likely due to the well-known action of organic matter, pH, and Ca on the reduction in metal mobility [32,33,38]. The similar or lower BCF values recorded in Penga Penga compared to Kalebuka (Figure 3) both support the hypothesis of reduced trace metal removal from tree rhizospheres in Penga Penga and reflect the likely existence of a mechanism to reduce metal uptake in a context of high trace metal exposure in the three species studied [39,40]. The results regarding the rhizosphere conditions support the use of mineral and organic amendments to ensure successful tree planting in Penga Penga. They also support the need for the long-term monitoring of metal concentrations after tree establishment to ensure the production of healthy fruit. 

### 4.2. Metal Accumulation in Leaves and Fruits

Below, the authors will discuss the results and how they can be interpreted from the perspective of previous studies and of the working hypotheses. The findings and their implications will be discussed in the broadest context possible. Future research directions will also be highlighted.

As expected, trace metal concentrations were generally higher in leaves and fruits harvested from Penga Penga compared to Kalebuka (Table 4), which can be explained by higher trace metal exposure as demonstrated in many woody species [40,41]. However, the concentration gaps are not proportional to those in the rhizospheres (Kalebuka vs. Penga Penga, see Table 3). For example, the Cu concentration was seven times higher in the rhizosphere in Penga Penga compared to Kalebuka, while it was similar in *S. guineense* leaves and 1.3 to 1.7 times higher in mango leaves and the fruits of both species (Table 4). As mentioned above, the use of amendments in the rhizospheres of plants in Penga Penga may explain the low concentrations in leaves and fruits. This hypothesis is supported by the BCF values (Figure 3), which were generally similar between the two districts despite the higher total concentrations of metals in the rhizosphere in Penga Penga. Indeed, variations in BCF values as a function of ecological conditions are documented [42] and would express, in the case of this study, exposure to different mobile concentrations of trace metals. In this context, the application of organic and mineral amendments would therefore be recommended to reduce metal accumulation in mango and *S. guineense* leaves and fruits in Penga Penga.

The results obtained also show an influence of the species on the accumulation of trace metals. Nevertheless, no clear trends emerged, except for As and Zn which seem to be accumulated in higher concentrations in mango leaves irrespective of the site. Future investigations should be undertaken to better identify trends, as this result may be important for the selection of trees to be grown according to organ consumption needs [43]. Furthermore, Cu and Zn were accumulated in higher concentrations regardless of species. This is most likely due to their higher concentrations in the polluted soil in Penga Penga [12,21] but accumulation was also seen in Kalebuka, as soils in the area have naturally higher concentrations of these two metals compared to the global average [9,22].

### 4.3. Human Exposure and Safe Weekly Consumption

Consistent with the concentration values in soils and plants, the results showed a higher proportion of values above the FAO and WHO limits in Penga Penga (Figure 2). In this case, the consumption of mango and *S. guineense* fruits and leaves may constitute an additional factor for exposure to trace metals, as already reported for other plant products in the region [7,44,45]. Nevertheless, calculations of the SWC showed that the consumption of *S. guineense* fruit is limited to 6.6 kg by the Cd concentration, while it is limited to 9 kg per week for mango flesh (and skin) by the Pb concentration (Table 5). These results led to two main conclusions. Firstly, these results show that it is not the most abundant elements in the polluted soil that are the most problematic in the context of Penga Penga but rather those that have a greater weight in terms of the health risk, as they are capable of causing serious diseases at low concentrations, as is the case for Cd and Pb [46,47,48]. Secondly, the recommended values of SWC are significant quantities, especially when considering on the one hand that the average mango weight is 0.3 kg to 0.4 kg (whole mango, kernel representing 10%) and the mean consumption varies from 2 to 3 mangoes per resident during the production period (September to December), and on the other hand that the average weight of a bunch of *Syzygium* is 0.27 to 0.42 kg and that the average consumption per resident is one bunch of fruit (personal observations). It can therefore be considered that the Penga Penga residents could safely consume mangoes within the limits proposed by the present study, as consumption of such a quantity does not represent a risk of intoxication if we consider the daily intake of the elements in the other nutrients. Furthermore, studies have reported a higher accumulation of trace metals in the skin of fruits, and mango in particular [49,50,51]. It would therefore be possible to increase the SWC value and recommend consumption quantities by removing the mango skin before consumption of the flesh. This would be a highly recommended change in habit for the residents of Penga Penga. 

With regard to leaves, their use in traditional medicine mixtures requires a more flexible interpretation. Indeed, the leaves of mango and *S. guineense* are not directly consumed after harvesting but rather subjected to boiling in order to migrate the desired active principles into the solution [52,53,54,55]. It is the leaf tea of these two species that is processed for consumption. In this context, although the FAO and WHO limited values provide an indication of the quality of the leaves produced, it would be awkward to propose safe consumption amounts. It would be preferable for future assessments to analyze the trace metal concentrations in the leaf teas of both species. In this way, more relevant recommendations could be suggested. However, the metal accumulation values in the leaves provide an idea of the exposure hazard and support the need for appropriate soil treatment to reduce the transfer of trace metals to the leaves. 

## 5. Conclusions

This study highlighted the soil mineral compositions in the rhizosphere of mango and *S. guineense* trees and their relative trace metal accumulation in leaves and fruits in the residential households of trace-metal-polluted soil from Penga Penga in comparison to Kalebuka, a non-polluted district. Furthermore, it provides recommendations on the amount of fruits that can be consumed safely by the residents. The results are encouraging for establishing fruiting trees in Penga Penga, as they demonstrate the positive impact of the planting protocol on the reduction in metal translocation into leaves and fruits. However, as almost fifty percent of samples from Penga Penga exceeded the FAO and WHO threshold values, it is suggested that agronomic practices should be improved to enhance the reduction in metal translocation into above-ground organs. In addition, it is recommended to test the removal of the skin on the SWI.

## Figures and Tables

**Table 1:** The pH, total organic C, and trace metal concentrations in the soil from two districts [22].

Parameters	Penga Penga	Kalebuka
pH KCl	6.3 (4.8–7.8)	6.8 (5.4–8.2)
TOC (%)	1.6 (0.5–3.0)	1.2 (0.4–0.2)
Co (mg.kg^−1^)	21 (4.6–90)	1.9–2.5
Cu (mg.kg^−1^)	2966 (213–17,096)	48 (45.3–50)
Zn (mg.kg^−1^)	202 (21–736)	18.2 (0–36.4)

**Table 2:** Safe weekly intake values for each metal analyzed in the study (mg per week). Calculated after PMTDI from the FAO and WHO [23] for As, Cd, Cu, and Pb, and from Pelkonen et al. [30] for Co and Zn.

Trace Metals	SWI (mg per Week)
As	1.26
Cd	0.35
Co	9.8
Cu	210
Pb	1.5
Zn	180

**Table 3:** The pH, major element, and trace metal concentrations of the surface soil (0–20 cm) collected in the rhizosphere of *M. indica* and *S. guineense* in Penga Penga and Kalebuka (Lubumbashi, DR Congo) (*n* = 60). Average (minimum–maximum) As_T_, Cu_T_, Pb_T_, and Zn_T_ = total As, Cu, Pb, and Zn concentrations; As_S_, Cd_S,_ Co_S_, Cu_S_, Pb_S,_ and Zn_S_ = soluble concentrations (extractable with 0.01 M CaCl_2_). References for uncontaminated soils around Lubumbashi (*n* = 18) from Shutcha et al. [9]. Means with different letters indicate significant differences (*p* < 0.05).

Parameters	Penga Penga	Kalebuka	References
pH	7.7 (6.7–8.4) a	6.4 (4.5–7.7) b	4.9–6.8
Al_2_O_3_ (%)	2.5 (1.4–5.0) a	3.0 (1.4–4.6) b	1.9–10.7
Fe_2_O_3_ (%)	4.3 (2.7–9.1) a	4.5 (2.5–6.5) a	0.9–7.4
Ca (%)	0.7 (0.1–2.3) a	0.3 (0.1–1.0) b	-
K (%)	0.9 (0.4–2.0) a	1.0 (0.5–1.6) a	-
Mn (mg.kg^−1^)	251 (89–679) a	292 (77–717) a	-
As_T_ (mg.kg^−1^)	12.8 (3–81) a	7.6 (3–16) b	-
Cu_T_ (mg.kg^−1^)	1379 (60–4670) a	189 (22–695) b	20–456
Pb_T_ (mg.kg^−1^)	142 (17–547) a	33 (2.0–110) b	7–82
Zn_T_ (mg.kg^−1^)	467 (129–1236) a	115 (31–275) b	26–180
As_S_ (mg.kg^−1^)	<0.01	<0.01	-
Cd_S_ (mg.kg^−1^)	0.07 (<0.01–0.5) a	0.13 (<0.01–1.05) a	-
Co_S_ (mg.kg^−1^)	0.31 (0.013–3.8) a	0.17 (0.01–1.7) a	-
Cu_S_ (mg.kg^−1^)	1.4 (0.2–7.4) a	0.7 (0.04–11.8) b	-
Pb_S_ (mg.kg^−1^)	0.11 (0.03–0.4) a	0.1 (0.06–0.4) a	-
Zn_S_ (mg.kg^−1^)	0.4 (0.01–6.9) a	1.7 (0.007–12.3) a	-

**Table 4:** Accumulation of metals (mg.kg^−1^) in leaves and fruits in Penga Penga (PP) and Kalebuka (KLB) (*n*= 60). Average (minimum–maximum) between Penga Penga and Kalebuka values. The values with the same letter are not significantly different (*p* < 0.05).

	Species	Leaves		Fruit		FAO/WHO Limits
		Penga Penga	Kalebuka	Penga Penga	Kalebuka	
As	*M. Indica*	0.2 a	0.1 b	0.1	<0.001	0.1
		(0.08–0.3)	(0.07–0.24)	(0.05–0.2)		
	*S. guineense*	0.1 a	0.05 b	0.02	<0.001	
		(0.07–0.2)	(0.04–0.08)	(0.00–0.1)		
Cd	*M. Indica*	0.1 a	0.14 a	0.19 a	0.09 b	0.2
		(0.07–0.3)	(0.05–0.22)	(0.1–0.3)	(0.01–0.4)	
	*S. guineense*	0.2 a	0.09 a	0.2 a	0.03 b	
		(0.06–0.8)	(0.03–0.3)	(0.1–0.3)	(0.02–0.05)	
Co	*M. Indica*	3.3 a	0.3 b	2.9 a	0.84 b	1
		(2.5–4.1)	(0.1–1.2)	(2.3–3.6)	(0.75–0.88)	
	*S. guineense*	2.7 a	0.7 b	0.84 a	0.79 a	
		(1.8–4.1)	(0.6–0.82)	(0.7–1.02)	(0.68–0.91)	
Cu	*M. Indica*	22.2 a	13 b	29.3 a	22.0 b	40
		(15–42)	(9–16)	(9–64)	(19–27)	
	*S. guineense*	18.4 a	19.8 a	18.9 a	15.9 b	
		(13–26)	(17–24)	(15–21)	(14–19)	
Pb	*M. Indica*	3.3 a	1.0 b	2.2 a	0.31 b	0.3
		(1.2–5)	(0.7–1.5)	(0.5–6)	(0.03–0.6)	
	*S. guineense*	2.2 a	0.8 b	0.8 a	0.11 b	
		(1.1–4.3)	(0.3–1.2)	(0.3–2.4)	(0.01–0.2)	
Zn	*M. Indica*	47.8 a	19.7 b	13.3 a	9.5 b	60
		(19–137)	(12–25)	(9–24)	(7–13)	
	*S. guineense*	20.3 a	13.9 b	39.1 a	14.7 b	
		(12–45)	(11–16)	(28–47)	(7–70)	

**Table 5:** Safe weekly consumption (kg per week) of *M. indica* and *S. guineense* fruits considering their metal concentrations for a 60 kg person in Penga Penga (PP) and Kalebuka (KLB) (*n* = 60). Safe weekly consumption was calculated after recommendations from the FAO and WHO [23]. Values are represented by the mean ± standard deviation; average (minimum–maximum).

Species	District	As	Cd	Co	Cu	Pb	Zn	Recommendation
*Mr. Indica*	PP	96 ± 61	17 ± 5	27 ± 3.5	89 ± 57	9 ± 6	119 ± 34	9
		(35–202)	(8–26)	(34–22)	(14–186)	(2–14)	(60–160)	
	KLB	20160	86 ± 103	93 ± 4.3	78 ± 10	80 ± 115	156 ± 26	78
		-	(6–280)	(105–89)	(14–186)	(2–14)	(60–160)	
*S. guineense*	PP	398 ± 253	6.6 ± 1.4	64 ± 7.3	61 ± 6.5	12 ± 6.4	21 ± 21	6.6
		(62–851)	(5.4–10)	(76–52)	(54–76)	(3.4–27)	(3.5–75)	
	KLB	1362	69 ± 53	68 ± 6.7	72 ± 6.2	161 ± 235	108 ± 38	68
		-	(38–210)	(60–78)	(60–81)	(35–810)	(14–139)	
SWI (mg per Week)		1.26	0.35	9.8	210	1.5	180	

## Data Availability

Not applicable.
